# Hemogram-Derived Ratios in the Prognosis of Acute Diverticulitis

**DOI:** 10.3390/medicina59091523

**Published:** 2023-08-23

**Authors:** Cristina Maria Sabo, Daniel-Corneliu Leucuta, Constantin Simiraș, Ioana Ștefania Deac, Abdulrahman Ismaiel, Dan L. Dumitrascu

**Affiliations:** 1 2nd Department of Internal Medicine, “Iuliu Hatieganu” University of Medicine and Pharmacy, 400006 Cluj-Napoca, Romania; cristina.marica90@yahoo.com (C.M.S.); abdulrahman.ismaiel@yahoo.com (A.I.); ddumitrascu@umfcluj.ro (D.L.D.); 2 Department of Medical Informatics and Biostatistics, “Iuliu Hatieganu” University of Medicine and Pharmacy, 400349 Cluj-Napoca, Romania; 3 Regional Institute of Gastroenterology and Hepatology, 400162 Cluj-Napoca, Romania; c_simiras@yahoo.com (C.S.); stefaniadeac24@gmail.com (I.Ș.D.)

**Keywords:** acute diverticulitis, Hinchey score, inflammatory markers, hemogram-derived ratios

## Abstract

*Background and Objectives*: It is crucial to quickly identify those patients who need immediate treatment in order to avoid the various complications related to acute diverticulitis (AD). Although several studies evaluated the neutrophil-to-lymphocyte ratio (NLR) suggesting its predictive value in assessing the severity of AD, results have been inconclusive. Therefore, we aimed to assess the relationship between the neutrophil-to-lymphocyte ratio (NLR), the platelet-to-lymphocyte ratio (PLR), the monocyte-to-lymphocyte ratio (MLR), and systemic immune inflammation (SII) with the severity of AD, the ability to predict the presence or absence of complications, and the recurrence rate, based on the values of inflammatory markers. *Materials and Methods*: We retrospectively reviewed 147 patients diagnosed with AD between January 2012 to February 2023. Patients were divided into 2 groups, uncomplicated and complicated AD. The characteristics and full blood count between both groups were compared. *Results:* A total of 65 (44.22%) patients were classified as having complicated AD. The area under the ROC curve (AUROC) defining a Hinchey score ≥ 1b was as follows: SII, 0.812 (95% confidence interval (CI), 0.73 –0.888); NLR, 0.773 (95% CI, 0.676–0.857); PLR, 0.725 (95% CI, 0.63–0.813); MLR: 0.665 (95% CI, 0.542 –0.777). An SII cutoff value of > 1200 marked the highest yield for diagnosing complicated AD, with a sensitivity of 82% and a specificity of 76%. The cumulative recurrence rate was not significantly different in the groups of SII ≥ median vs. SII < median (*p* = 0.35), NLR ≥ median vs. NLR < median (*p* = 0.347), PLR ≥ median vs. PLR < median (*p* = 0.597), and MLR ≥ median vs. MLR < median (*p* = 0.651). *Conclusions*: Our study indicates that SII, NLR, and PLR are statistically significant and clinically useful classifying ratios to predict higher Hinchey scores. However, they cannot predict recurrences.

## 1. Introduction

Acute diverticulitis (AD) is defined as an inflammation of the diverticula and can be either uncomplicated or complicated. Around 5% of patients with diverticulosis will experience at least one episode of diverticulitis during their lifetime [1]. Most cases of AD (75%) are uncomplicated and are characterized by the presence of limited inflammation to the wall of the colon, with or without an inflammatory phlegmon confined to the colonic wall [2]. It is a relatively mild disease and one that can be safely treated in primary care [3]. Only 12% of patients with acute diverticulitis will present with complications. Among the complications, the most frequent is the presence of an abscess, followed by peritonitis, obstruction, and fistula [4]. Higher mortality rates were found among patients with complicated AD [4].

Numerous factors have been linked to a higher probability for developing AD. These factors include age [4], sex [4], genetics [5,6,7,8], adherence to a Western dietary pattern (characterized by the high consumption of red meat, high-fat dairy, and refined grains) [9], smoking [10], obesity [11,12,13], and the use of certain medications (such as nonsteroidal anti-inflammatory drugs [NSAIDs], opiate analgesics, and corticosteroids) [14].

The triad of symptoms consisting of left-sided lower abdomen discomfort, lack of vomiting, and a C-reactive protein (CRP) levels greater than 5 mg/dl, shows a high sensitivity for detecting AD (acute diverticulitis). However, this triad does not allow differentiating between uncomplicated and complicated diverticulitis [5]. The World Society of Emergency Surgery (WSES) recommends a comprehensive evaluation of patients, including clinical history, physical signs, laboratory inflammation markers, and radiological findings. The first choice of imaging technique is a contrast-enhanced computed tomography (CT) scan of the abdomen, or a step-up approach with CT being performed after an inconclusive or negative ultrasound (US) in patients suspected of having AD [6]. Unfortunately, this approach may lead to a significant number of patients with uncomplicated diverticulitis being referred to the emergency department, resulting in unnecessary CT scans.

Circulating cells, such as lymphocytes, monocytes, neutrophils, and platelets are essential components of the inflammatory process. Their ratios, comprising the neutrophil-to-lymphocyte ratio (NLR), platelet-to-lymphocyte ratio (PLR), monocyte-to-lymphocyte ratio (MLR), and systemic immune inflammation (SII), are simple and easily accessible inflammatory markers that have been studied as potential prognostic indicators in various medical conditions. Previous studies have found that high NLR and PLR values have been linked with complicated diverticulitis [7,8], surgical intervention [9], the failure of conservative treatment for acute first-attack colonic diverticulitis [10], shorter intervals between recurrent episodes, and longer cumulative hospitalization days [11]. The monocyte-to-lymphocyte ratio (MLR) and systemic immune inflammation (SII) have been studied as potential prognostic markers in coronavirus disease, cardiovascular diseases, sepsis, and cancer. Moreover, one study assessed their correlation with the failure of conservative management [12].

The development of a diagnostic and prognostic tool to identify complicated AD and assess the risk of complications in patients with uncomplicated AD can indeed be beneficial in reducing healthcare costs, unnecessary referrals, and the excessive use of CT scans. 

Therefore, the objective of this study was to evaluate the predictive value of NLR, PLR, MLR, and SII regarding to the severity of AD, their ability to predict the presence or absence of complications, and the recurrence rate based on inflammatory marker values.

## 2. Materials and Methods

### 2.1. Study Desing, Setting, and Participants

We conducted a retrospective observational study involving subjects ≥18 years old. All patients who were admitted with acute diverticulitis (AD) at the Clinical Emergency County Hospital of Cluj-Napoca, Romania between January 2012 to February 2023 were identified, and their data were collected retrospectively using electronic medical records. The patients were searched for in the hospital database, using the ICD (International Classification of Disease) code K57 (diverticular disease of the intestine). All included patients in our study were diagnosed with AD using either a CT scan, colonoscopy, or medical/surgical report. Patients were excluded from the study based on the following criteria: (1) any malignancy, (2) a lack of confirmation of AD, and (3) a lack of laboratory parameters (leukocyte, neutrophils, lymphocytes, platelets, or monocytes) at admission. 

The study was conducted according to the principles of the Declaration of Helsinki and was approved by the Ethical Committee of “Iuliu Hațieganu” University of Medicine and Pharmacy Cluj-Napoca (approval no. 139/27.06.2023).

The research has been conducted following the Strengthening the Reporting of Observational Studies in Epidemiology (STROBE) guidelines [15]. The comprehensive STROBE checklist can be found in Table S1.

### 2.2. Variables of Interest

The primary outcomes were to evaluate the NLR, PLR, MLR, and SII levels in patients with complicated AD and to investigate the diagnostic and prognostic accuracy of these markers.

### 2.3. Data Sources and Measurements

We collected the following information from patient medical records: age, sex, body mass index (BMI), clinical symptoms at admission (abdominal pain, nausea, vomiting, constipation, diarrhea, and fever), laboratory parameters, diagnosis method (CT, colonoscopy, or surgical/medical reports), length of hospital stay, recurrence rate, the time interval from the first to a recurrent episode, cumulative length of stay due to AD, and initial treatment strategy (such as careful observation, antibiotic treatment, abscess drainage or surgery, and type of intervention). Reviewing of the records was performed by three investigators (C.M.S., I.D., and S.C.).

The diagnosis of AD was based on the presence of inflammation in one or more diverticula based on CT scans, surgical reports, or colonoscopy. AD can be either uncomplicated or complicated. The classification used to define AD severity was the modified Hinchey classification based on CT findings [1]. The modified Hinchey score was as follows: stage 0—mild clinical diverticulitis; stage 1A—confined pericolic inflammation or phlegmon; stage 1B—confined pericolic abscess; stage 2—pelvic, intraabdominal, or retroperitoneal abscess; stage 3—generalized purulent peritonitis; and stage 4—fecal peritonitis [1].

Laboratory parameters included leukocytes, neutrophils (N), lymphocytes (Ly), monocytes (M), platelets (P), red cell distribution width (RDW), platelet distribution width (PDW), urea, and creatinine. NLR (the neutrophil-to-lymphocyte ratio) and PLR (the platelet-to-lymphocyte ratio) were defined as neutrophil or platelet counts divided by the total number of lymphocytes. MLR (the monocyte to lymphocyte ratio) was defined as the absolute monocyte count, divided by the absolute lymphocyte count. The systemic immune-inflammatory index (SII) was calculated using the formula SII = (P × N)/L.

The number of cases with AD diagnosis in our hospital during the study period determined the sample size. We grouped the patients into two categories of uncomplicated (0, IA) vs. complicated diverticulitis (1B–4). Moreover, for a more detailed analysis, the patients were further divided into three groups according to the modified Hinchey grade. Patients with a modified Hinchey score of 0 or IA were considered the uncomplicated AD group, while patients with a modified Hinchey score of IB or II were diagnosed as having complicated AD with an abscess, and patients with modified Hinchey score of III were diagnosed as belonging to the complicated AD with perforation group.

### 2.4. Statistical Analysis

Data were presented as counts and percentages when the variables were categorical, as means and standard deviations when the variables were quantitative and with a normal distribution, or as medians and interquartile ranges when the variables were quantitative and did not follow a normal distribution. The comparisons between two independent groups concerning categorical data were conducted using a chi-squared test or Fisher’s exact test. For quantitative data that did not follow a normal distribution, we used the Mann-Whitney U test. For quantitative data that followed a normal distribution, the analysis was performed using an independent *t*-test. Comparisons between three independent groups concerning quantitative data that did not follow a normal distribution were made using the Kruskal–Wallis method. The discriminating qualities of inflammatory markers were assessed using receiver operating characteristic (ROC) curves, using their area under the curve (with a 95% bootstrapped computed confidence interval [CI]), as well as by identifying the best cutoff point by maximizing the Youden index and computing its sensitivity and specificity. Comparisons between ROC curves were performed with the De Long test. The intervals between episodes were compared using Kaplan–Meier survival curves, and the Cox proportional hazards regression model was used to analyze associations of several factors such as NLR, PLR, MLR, and SII with cumulative recurrence rate. For all statistical tests, a two-tailed *p*-value was computed, and a value of ≤0.05 was considered to be statistically significant. All analyses were computed in the R environment for statistical computing and graphics, version 4.1.2 (R Foundation for Statistical Computing, Vienna, Austria).

## 3. Results

### 3.1. General Characteristics

The cohort included 147 patients, of which, 71 (49.98%) patients were females. The mean age was 60.8 years, ranging from 25–89 years. Only 23% of the patients (*n* = 34) were younger than 50 years of age. According to the modified Hinchey classification [13,14], 70 (47.61%) patients were diagnosed with uncomplicated acute diverticulitis, and 57 (38.77%) were diagnosed with complicated disease. Of the complicated diverticulitis group, 44 (29.93%) patients showed CT findings that were compatible with Hinchey stage III disease, and none with Hinchey stage IV disease. The demographic and clinical characteristics of the subjects are presented in Table 1.

### 3.2. Patients’ Characteristics According to the Severity of Diverticulitis

The patients were categorized into two groups based on the presence or absence of complications. The characteristics of patients between uncomplicated AD and complicated AD are compared in Table 1. A total of 65 subjects were diagnosed with complicated acute diverticulitis and 82 subjects with uncomplicated acute diverticulitis. Compared to uncomplicated AD, complicated AD patients presented statistically significant differences in terms of the location of the diverticula in the sigmoid colon (*n* = 47 (82.46%) vs. *n* = 38 (54.29%), *p* < 0.001), the presence of diffuse abdominal pain (*n* = 24 (38.1%) vs. *n* = 13 (15.85%), *p* = 0.002) and constipation (*n* = 3 (4.76%) vs. *n* = 14 (17.07%), *p* = 0.022), as well as the treatment. There was no significant difference between uncomplicated and complicated AD groups in other factors, such as age, sex, abdominal pain, diarrhea, nausea, vomiting, and fever. 

### 3.3. Inflammatory Markers in Patients with AD

The patients were classified into three groups, based on the Hinchey classification, namely the O/IA group (uncomplicated AD) (*n* = 82), the IB/II group (complicated AD with abscess) (*n* = 21), and the III group (complicated AD with perforation) (*n* = 44). We compared total and differential white blood cell (WBC) counts, along with six novel inflammatory ratios in uncomplicated AD patients, complicated AD patients with an abscess, and complicated AD with perforation, as demonstrated in Table 2 and Table 3. These inflammatory ratios included MLR, NLR, PDW, PLR, RDW, and SII.

Firstly, the WBC (*p*-value < 0.001), neutrophil (N) (*p*-value < 0.001), and platelet (P) (*p*-value < 0.001) values were significantly different between the three groups. Secondly, three inflammatory ratios demonstrated a statistically significant difference between the three groups, including NLR (*p*-value < 0.001), PLR (*p*-value < 0.001), and SII (*p*-value <0.001). Comparing every two groups, WBC, N, P, NLR, PLR, and SII were significantly different between the uncomplicated AD group and the complicated AD with abscess group, and no statistically significant difference between complicated AD with abscess group and the complicated AD with perforation group. These values, except in the case of platelets, were significantly higher in patients with complicated AD with perforation, compared to those with uncomplicated AD group.

**Figure 1:**
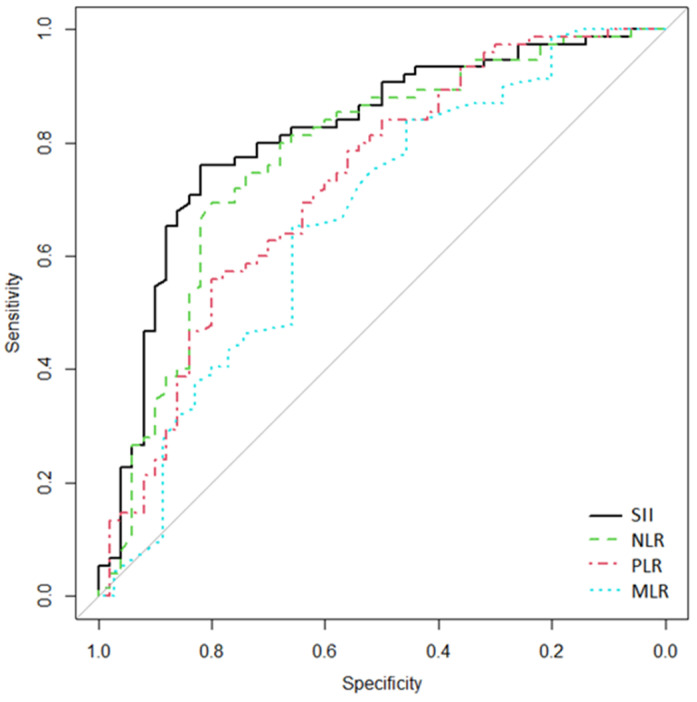
Receiver operating characteristics (ROC) curves for SII, NLR, PLR, and MLR to discriminate the complicated AD group (modified Hinchey grade > IA). The *x*-axis shows false-positive/1-specificity, and on the *y*-axis, true positive/sensitivity is expressed.

The above results showed that SII, NLR, and PLR levels were significantly higher in the complicated AD group (modified Hinchey grade of > IA). We studied the cutoff point, the area under the curve (AUC), Se, and Sp for these inflammatory ratios (Table 4). The ROC predicting curve analysis (Figure 1) showed an AUC and 95% CI of SII, NLR, PLR, and MLR in predicting complicated AD of 0.812 (95% CI [0.73–0.888]), 0.773 (95% CI [0.676 –0.857]), 0.725 (95% CI [0.63–0.813]), 0.665 (95% CI [0.542–0.777]), respectively. The diagnostic value of SII was the highest. When the cutoff value of SII was 1200, the sensitivity and specificity were 82% and 76%, respectively. There was no significant difference in AUC compared with each other (*p* > 0.05) (Table 4).

**Figure 2:**
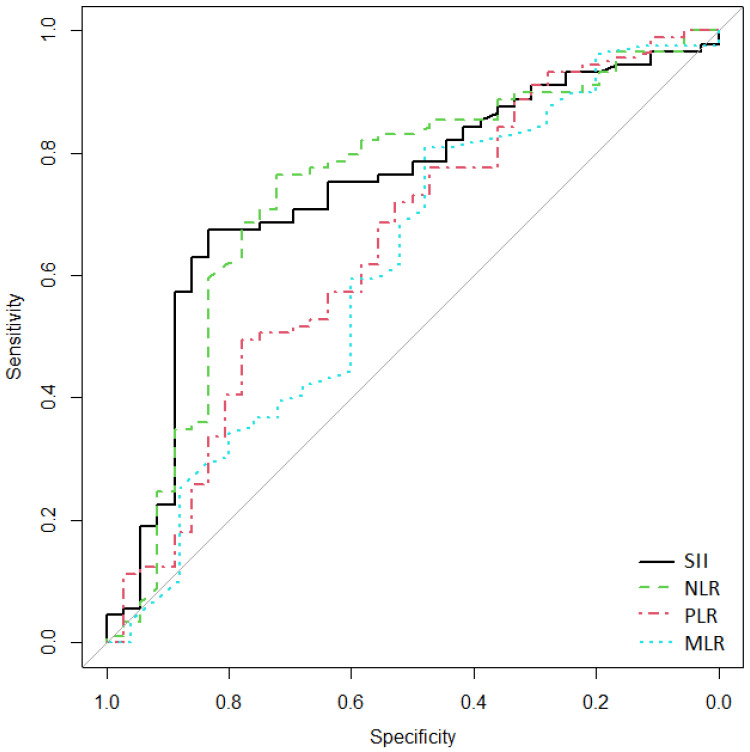
Receiver operating characteristics (ROC) curves for SII, NLR, PLR, and MLR, used to discriminate cases of complicated AD with perforation (modified Hinchey grade III). On the *x*-axis, false-positive/1-specificity is shown, and on the *y*-axis, true positive/sensitivity is expressed.

The ROC curve (Figure 2) showed that the AUC of SII, NLR, PLR, and MLR in the diagnosis of complicated AD with perforation (modified Hinchey grade III) were 0.738 (95% CI [0.632–0.831]), 0.737 (95% CI [0.629–0.834]), 0.65 (95% CI [0.53–0.754]), and 0.614 (95% CI [0.474–0.738]), respectively. The diagnostic value of SII was the highest. When the cutoff value of SII was 1200, the sensitivity and specificity were 83.3% and 67.4%, respectively (Table 5).

**Figure 3:**
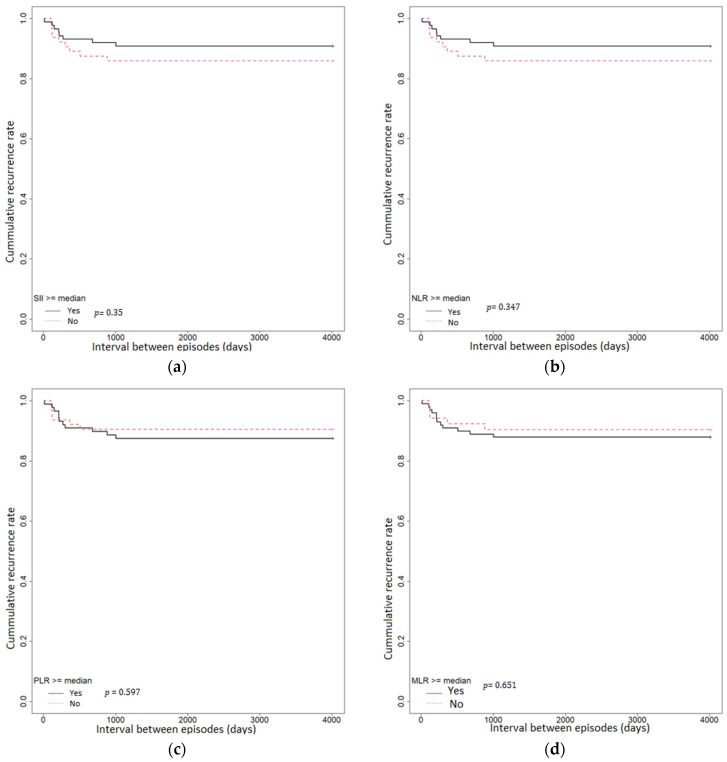
Kaplan–Meier analysis of the intervals between episodes of AD: (**a**) among patients with SII ≥ median (**b**) among patients with an NLR ≥ median; (**c**) among patients with a PLR ≥ median; (**d**) among patients with an MLR ≥ median.

Additional episodes of acute diverticulitis occurred in 15 (10.20%) patients; of these, 4 (26.66%) patients presented more than one episode of acute diverticulitis. Survival analysis was used to assess the predictive value of SII, NLR, PLR, and MLR regarding the intervals between episodes. The intervals between episodes were compared using Kaplan–Meier survival curves (Figure 3). Using the log-rank test, there were no statistically significant differences in the cumulative recurrence rate between SII ≥ median (=1101.57) vs. SII < median group (*p* = 0.35), NLR ≥ median (=4.06) vs. NLR < median (*p* = 0.347), PLR ≥ median (=168.64) vs. PLR < median (*p* = 0.597), and MLR ≥ median (=0.36) vs. MLR < median (*p* = 0.651) in patients with acute diverticulitis. Using the Cox proportional hazards regression model, we observed that the interval between episodes was higher in the PLR < median group and MLR < median group compared with PLR ≥ median group and MLR ≥ median group. However, it was lower in the NLR < median group and SII < median group compared with the NLR ≥ median group and SII ≥ median group, albeit not being statistically significant (Table 6).

## 4. Discussion

It is crucial to recognize complicated AD at an early admission stage for prognosis, risk assessment, the allocation of resources, appropriate disease management, and informed clinical decision-making. This allows for tailored interventions based on the severity of the disease and helps optimize patient outcomes. A CT scan is the gold standard of diagnostic tools for acute diverticulitis since it is, effective in evaluating its severity, and excluding alternative diagnoses. To optimize the use of CT scans, it is recommended to consider the clinical and laboratory findings when dealing with suspected complicated diverticulitis. This approach helps minimize the treatment expenses and potential radiation risks.

This retrospective study assessed the associations between NLR, PLR, MLR, and SII regarding the severity of acute diverticulitis. The values for these hemogram-derived ratios were easily and quickly obtained. Moreover, we also evaluated the ability of these ratios to predict the presence of complications and the disease recurrence rate, based on these inflammatory ratio values. We included a total population of 147 patients who were admitted with acute diverticulitis, out of whom 44.2% (*n* = 65) of patients presented with complicated AD. Firstly, we observed that the SII, NLR, and PLR levels were significantly higher in the complicated AD group (modified Hinchey grade >IA), but no statistically significant difference was reported between the complicated AD with abscess group and the complicated AD with perforation group. Secondly, the SII, NLR, and PLR were the most accurate inflammatory ratios associated with complicated AD. Lastly, we did not observe a correlation between the SII, NLR, and PLR levels and the interval between episodes of acute diverticulitis. To the best of our knowledge, this is the first study to evaluate the utility of the SII and MLR levels for diagnosing AD.

The mean age in our study was 60.8 years, with patients ranging from 25 to 89 years. Only 23% were younger than 50 years of age. According to the World Society of Emergency Surgery (WSES) guidelines [16], caution is advised against solely relying on the patient’s clinical signs, symptoms, and laboratory tests for diagnosing left colonic AD in the elderly population. To confirm the diagnosis and distinguish between complicated and uncomplicated AD in elderly patients, the guidelines recommend using a CT scan with IV-contrast dye. Alternatively, when intravenous contrast enhanced CT scan is not feasible, ultrasound (US), magnetic resonance imaging (MRI), or a CT scan without IV contrast dye may be considered based on resource availability.

The prevalence of complicated AD in our study was similar to that reported by Zager et al. [11] (44.2% (*n*= 65) vs. 32.7% (*n* = 149]), but was different from that reported by Palacios Huatuco et al. [17] and Bharucha et al. [4] (44.2% (*n* = 65) vs. 9 % (*n* = 30) vs. (12% (*n* = 386)). In the complicated diverticulitis group, 29.93% (*n* = 44) of patients had CT findings compatible with a diagnosis of Hinchey stage III. Recurrent episodes of acute diverticulitis occurred in 10.20% (*n* = 15) of patients, of whom 26.66% (*n* = 4) presented more than one episode of acute diverticulitis. We also found that patients with complicated AD were of a similar age to those with uncomplicated AD (median age, 59.22 vs. 62.05 years, *p* = 0.226). These findings are similar to those reported by Palacios Huatuco et al. [17] and are different from those reported by Chang et al. [18].

In our study, WBC, neutrophil, platelet, and monocyte counts were higher in the complicated AD group (Hinchey IB/II and Hinchey III), while lymphocytes were lower in this group, suggesting an inflammatory response and immune system activation. Only WBC, neutrophil, and platelet counts have a statistically significant association with complicated AD. However, we did not find any statistically significant differences related to these values in the group with perforation compared to those with abscesses, as we would have expected. This unexpected finding may indicate that other factors beyond these specific markers play a significant role in differentiating between these two types of complications in AD.

Circulating cells, such as lymphocytes, monocytes, neutrophils, and platelets are essential components of the inflammatory process. In contrast to measuring each WBC component separately, measuring the divergence among them has been thought to be more accurate for predicting poor clinical outcomes [19]. Previous studies that have used two hemogram-derived ratios, namely, the NLR and PLR, have found that high NLR or PLR have been linked with complicated diverticulitis [7,8], surgical intervention [9], the failure of conservative treatment for acute first-attack colonic diverticulitis [10], a shorter interval between recurrent episodes, and longer cumulative hospitalization days [11]. Several studies have studied MLR and SII as potential prognostic markers in coronavirus disease, cardiovascular diseases, sepsis, and cancer; however, one study has assessed their correlation with the failure of conservative disease management [12]. In our cohort, we found that SII, NLR, and PLR levels were significantly higher in the complicated AD group (modified Hinchey grade >IA), but the MLR level was not significantly different between the uncomplicated AD and complicated AD groups.

In our study, the SII cutoff of 1200 had a sensitivity of 82% and a specificity of 76%; the NLR cutoff of 4.06 had a sensitivity of 80% and a specificity of 69.3%; and the PLR cutoff of 144.38 had a sensitivity of 80% and a specificity of 56%. The diagnostic accuracy of the SII, NLR, and PLR for complicated AD is good and is also comparable, but MLR could only predict those patients who had complicated AD with low accuracy (AUC = 0.665, Se = 65.7%, Sp = 65.2%). The SII showed the highest AUROC value of 0.812, indicating its ability to discriminate effectively between patients with and without complicated AD. This finding is particularly interesting since the SII showed a diagnostic potential similar to that of procalcitonin, a well-known biomarker for infection and inflammation [20]. Regarding the cutoff values for the NLR and PLR, we found that the reported values in the literature align with our observations. The cutoff values for the NLR and PLR vary according to the specific clinical context. In cases of malignancy, the cutoff value of NLR typically hovers around 3, while sepsis situations tend to require a higher cutoff value of approximately 5 [21]. In our study, we found a NLR cutoff value of 4.06, which is within the range reported for sepsis situations. This suggests that NLR may be a relevant marker for distinguishing sepsis-related conditions, such as complicated acute diverticulitis. This finding aligns with a study by Palacios Huatuco et al. [17] who found a value of NLR 4.2 (Se: 80%, Sp: 64.1%), with the best diagnostic yield to define severity. The cut-off value of above 150 for PLR is widely reported and published by various authors both in oncology [22] and sepsis conditions [23]. In our study, we calculated a PLR cutoff value of 144.38, which is remarkably close to the reported value. This correspondence with the internationally reported cutoff value strengthens the validity of our study and endorses the reliability of PLR as a potential diagnostic marker for complicated AD. Mari et al. found that the PLR demonstrated lower diagnostic accuracy than the NLR (AUC values of 0.73, and 0.77, respectively) [7]. Zager et al. did not find an independent association between a high PLR (>120) and complicated disease [11]. The PLR cutoff value of 120 used by Zager et al. might have been lower than the optimal threshold that is required to detect an independent association with complicated disease. Park et al. [12] showed that NLR, lymphocyte-to-monocyte ratio (LMR), PLR, and SII levels were not predictive factors in ascertaining the success or failure of conservative management in patients with right colonic diverticulitis.

In a previous study conducted by Zager et al. [11], it was reported that elevated levels of NLR (>5.4) were associated with shorter intervals between episodes of acute diverticulitis, an increased number of readmissions, and a longer hospital stay. In our study, we did not find statistically significant differences in the cumulative reoccurrence rates between the groups based on SII, NLR, PLR, and MLR. This suggests that these inflammatory ratios may not be strongly associated with the interval between episodes of acute diverticulitis. 

Our research has several limitations. First, the data were collected retrospectively; hence, a selection bias may exist, and also, some medical records did not contain all variables. Second, this was a single-center study conducted in Eastern Europe with a relatively small sample size, which may affect the statistical power of our findings and limit their generalizability to other populations or settings. Third, smoking status, medication use, and BMI could not be included in the analysis due to lack of data. Most patients with diverticulosis are obese and are diagnosed concurrently with metabolic dysfunction-associated steatotic liver disease (MASLD) [24]. It has been observed that overweight and obesity are associated with an increased risk of moderate/severe diverticular disease, complicated diverticular disease with abscesses or perforation, the need for emergency surgical intervention, and diverticular bleeding in both sexes. A recent meta-analysis, which included six studies, calculated a relative risk (RR) of 1.31 (95% CI 1.09–1.56) and 1.20 (95% CI 1.04–1.40) for diverticular disease and complicated diverticular disease, respectively [25]. The presence of obesity and metabolic syndrome was not determined in our study cohort due to the lack of data in each patient’s medical records. Obesity influences NLR; therefore, the absence of this data represents another limitation of the current study.

However, despite these limitations, our study also has several important strengths. It provides a valuable and comprehensive evaluation of multiple inflammatory markers and hemogram-derived ratios in complicated AD. It is the first study to evaluate the utility of SII and MLR for diagnosing complicated AD and establish their prognostic utility. Additionally, the associated complications were accurately evaluated using CT scan or surgical report. 

We believe that the clinical significance of this study lies in its potential to improve diagnostic accuracy, guide treatment decisions, optimize healthcare resource utilization, and contribute to the existing knowledge in the field of complicated acute diverticulitis. By evaluating multiple inflammatory markers, including SII and MLR, this study expands the range of diagnostic tools available for complicated acute diverticulitis, enhancing the accuracy and efficiency of diagnosing the severity of the disease. The early recognition of complicated AD allows for timely and targeted interventions, such as performing additional imaging studies or initiating more intensive monitoring, guiding decisions about the need for surgical consultation or aggressive medical management. Furthermore, by ruling out complicated AD in a considerable proportion of cases with good specificity, the inflammatory parameters can help avoid unnecessary CT scans and hospitalizations, thus optimizing healthcare resource utilization. Finally, it fills a gap in the literature by being the first to evaluate the utility of SII and MLR in diagnosing complicated acute diverticulitis, expanding the understanding of inflammatory markers in this specific context and opening avenues for further research and validation.

Therefore, future studies with larger sample sizes, multi-center designs, and prospective data collection methods are needed to confirm our results on the predictive role of hemogram-derived ratios in complicated AD and to refine the optimized cutoff values for the assessed parameters.

## 5. Conclusions

SII, NLR, and PLR can predict complicated AD, with an SII of >1200 having the highest yield for diagnosing complicated AD. Incorporating these hemogram-derived ratios into a comprehensive predictive model may improve accuracy and aid in clinical decision-making for patients with AD.

## Figures and Tables

**Table 1:** Demographic characteristics of patients with acute diverticulitis.

Characteristic	Number (%) (*n* = 147)	Uncomplicated AD (*n* = 82)	Complicated AD *(n* = 65)	*p*-Value
**Age (years), mean (SD)**	60.8 (14.05)	62.05 (14)	59.22 (14.06)	0.226
**Sex**				
female	72 (48,97)	40 (57.14)	32 (56.14)	0.957
**Location of diverticula**				
rectum	1 (0.79)	1 (1.43)	0 (0)	1
sigmoid colon	85 (66.93)	38 (54.29)	47 (82.46)	<0.001
descending colon	13 (10.24)	6 (8.57)	7 (12.28)	0.493
transverse colon	4 (3.15)	4 (5.71)	0 (0)	0.127
ascending colon	3 (2.36)	2 (2.86)	1 (1.75)	1
cecum	0 (0)			
**Symptoms**				
abdominal pain	133 (91.72)	72 (87.8)	61 (96.83)	0.051
right iliac region	10 (6.9)	5 (6.1)	5 (7.94)	0.747
right lumbar region	4 (2.76)	3 (3.66)	1 (1.59)	0.633
right hypochondriac region	3 (2.07)	3 (3.66)	0 (0)	0.258
epigastric region	3 (2.07)	3 (3.66)	0 (0)	0.258
left hypochondriac region	5 (3.45)	5 (6.1)	0 (0)	0.069
left lumbar region	30 (20.69)	20 (24.39)	10 (15.87)	0.209
left iliac region	63 (43.45)	38 (46.34)	25 (39.68)	0.423
hypogastric region	19 (13.1)	9 (10.98)	10 (15.87)	0.386
umbilical region	1 (0.69)	1 (1.22)	0 (0)	1
diffuse	37 (25.52)	13 (15.85)	24 (38.1)	0.002
diarrhea at admission	43 (29.66)	29 (35.37)	14 (22.22)	0.086
constipation at admission	17 (11.72)	14 (17.07)	3 (4.76)	0.022
nausea	19 (13.1)	12 (14.63)	7 (11.11)	0.533
vomiting	17 (11.72)	8 (9.76)	9 (14.29)	0.401
fever	27 (18.62)	11 (13.41)	16 (25.4)	0.066
**Hinchey classification**				
stage 0	43 (29.25)			
stage IA	39 (26.53)			
stage IB	10 (6.8)			
stage II	11 (7.48)			
stage III	44 (29.93)			
stage IV	0 (0)			
**Treatment**				
conservative	106 (72.11)	80 (97.56)	26 (40)	<0.001
drainage	22 (14.97)	1 (1.22)	21 (32.31)	<0.001
surgery				
without	103 (70.07)	80 (97.56)	23 (35.38)	
emergency	37 (25.17)	1 (1.22)	36 (55.38)	<0.001
elective	7 (4.76)	1 (1.22)	6 (9.23)	
**Recurrence**				
one episode	12			
two episodes	2			
≥ three episodes	1			
unknown	1			

SD, standard deviation; AD, acute diverticulitis.

**Table 2:** Total and differential leukocyte counts in the uncomplicated and complicated AD groups.

Hinchey Classification	0/Ia (*n* = 82)	Ib/II (*n* = 21)	III (*n* = 44)	*p*-Value
WBC (×10^9^/mL), median (IQR)	7.95 (6.45–9.84)	11.11 (9.67–16.34)	11.18 (7.29–13.26)	<0.001 (0.003/0.01/0.742) [*n*1 = 79, *n*2 = 20, *n3* = 43]
WBC, *n* (%)				< 0.001
Below NR	0 (0)	0 (0)	2 (4.65)	
NR	64 (81.01)	10 (50)	19 (44.19)	
Above NR	15 (18.99)	10 (50)	22 (51.16)	
Ly (×10^9^/mL), median (IQR)	1.6 (1.24–2)	1.35 (1.05–1.72)	1.44 (0.9–1.76)	0.096 (0.336/0.233/1) [*n*1 = 75, *n*2 = 14, *n*3 = 36]
Ly, *n* (%)				0.012
Below NR	9 (12)	3 (21.43)	13 (36.11)	
NR	66 (88)	11 (78.57)	23 (63.89)	
M (×10^9^/mL), median (IQR)	0.47 (0.37–0.71)	0.76 (0.52–0.92)	0.55 (0.4–0.74)	0.169 (0.163/0.85/0.523) [*n*1 = 69, *n*2 = 10, *n*3 = 25]
M, *n* (%)				0.017
Below NR	0 (0)	0 (0)	2 (8)	
NR	58 (84.06)	5 (50)	18 (72)	
Above NR	11 (15.94)	5 (50)	5 (20)	
N (×10^9^/mL), median (IQR)	5.38 (4.16–7.36)	8.69 (6.96–12.3)	9.48 (5.38–11.96)	<0.001 (0.004/0.009/0.983) [*n*1 = 75, *n*2 = 14, *n*3 = 36]
N, *n* (%)				<0.001
Below NR	1 (1.33)	0 (0)	1 (2.78)	
NR	54 (72)	4 (28.57)	13 (36.11)	
Above NR	20 (26.67)	10 (71.43)	22 (61.11)	
P (×10^3^/mL), median (IQR)	239 (195.5–273.5)	300 (272.5–447.5)	257 (227–354)	<0.001 (<0.001/0.175/0.051) [*n*1 = 79, *n*2 = 20, *n*3 = 42]
P, *n* (%)				0.01
Below NR	4 (5.06)	0 (0)	4 (9.52)	
NR	72 (91.14)	15 (75)	31 (73.81)	
Above NR	3 (3.8)	5 (25)	7 (16.67)	

IQR, interquartile range; NR, normal range; WBC, white blood cell (NR = 4–11); Ly, lymphocyte (NR = 1–4.8); M, monocytes (NR = 0.2–0.8); N, neutrophil (NR = 2.5–7); P, platelet (NR = 150–450); values between the square brackets represent the number of observations per group.

**Table 3:** Inflammatory ratio values in the uncomplicated and complicated AD groups.

Hinchey Classification	0/Ia (*n* = 82)	Ib/II (*n* = 21)	III (*n* = 44)	*p*-Value
MLR, median (IQR)	0.35 (0.21–0.42)	0.43 (0.4–0.6)	0.42 (0.28–0.62)	0.013 (0.101/0.133/0.738) [*n*1 = 69, *n*2 = 10, *n*3 = 25]
NLR, median (IQR)	3.53 (2.51–4.81)	5.86 (4.14–13.18)	6.96 (5.1–10.52)	<0.001 (0.006/<0.001/0.976) [*n*1 = 75, *n*2 = 14, *n*3 = 36]
PDW, median (IQR)	13.75 (11.83–16)	12.9 (10.75–15.75)	13.95 (11.12–15.9)	0.318 (0.383/0.614/0.767) [*n*1 = 70, *n*2 = 16, *n*3 = 40]
PLR, median (IQR)	140.15 (108.1–214.34)	278.32 (204.28–426.21)	228.7 (149.68–400.46)	<0.001 (0.002/0.006/0.541) [*n*1 = 75, *n*2 = 14, *n*3 = 36]
RDW, median (IQR)	42.3 (40.1–46.6)	44.65 (41.18–50.9)	43.3 (41.5–46.2)	0.443 (0.584/0.755/0.782) [*n*1 = 65, *n*2 = 12, *n*3 = 29]
SII, median (IQR)	773.24 (539.28–1173.68)	2856.29 (1434.65–3631.29)	1689.24 (1310.22–3532.76)	<0.001 (<0.001/<0.001/0.775) [*n*1 = 75, *n*2 = 14, *n*3 = 36]

IQR, interquartile range; NLR, neutrophil-to-lymphocyte ratio; PLR, platelet-to-lymphocyte ratio; MLR, monocyte-to-lymphocyte ratio; SII, systemic immune inflammation; PDW, platelet distribution width; RDW, red blood cell distribution width; values between square brackets represent the number of observations per group.

**Table 4:** The area under the receiver operating characteristic (ROC) curve (AUC) and cutoff values for MLR, NLR, PLR, and SII in the complicated AD (modified Hinchey grade > IA), as well as comparisons between the ROC curves.

Characteristic	AUROC (95% CI)	Cutoff	Se	Sp
MLR	0.665 (0.542–0.777)	0.38	65.7	65.2
NLR	0.773 (0.676–0.857)	4.06	80	69.3
PLR	0.725 (0.63–0.813)	144.38	80	56
SII	0.812 (0.73–0.888)	1200	82	76
SII vs. NLR	*p *= 0.111			
SII vs. MLR	*p *= 0.027			
SII vs. PLR	*p *= 0.019			
NLR vs. PLR	*p *= 0.323			

AUROC, the area under the receiver operating characteristic curve; CI, confidence interval; NLR, neutrophil-to-lymphocyte ratio; PLR, platelet-to-lymphocyte ratio; MLR, monocyte-to-lymphocyte ratio; SII, systemic immune inflammation; Se, sensitivity; Sp, specificity.

**Table 5:** The area under the ROC curve (AUROC) for MLR, NLR, PLR, and SII in the complicated AD with perforation group (modified Hinchey grade III).

Characteristic	AUROC (95% CI)	Cutoff	Se	Sp
MLR	0.614 (0.474–0.738)	0.51	48	81
NLR	0.737 (0.629–0.834)	5.61	72.2	76.4
PLR	0.65 (0.53–0.754)	144.38	77.8	49.4
SII	0.738 (0.632–0.831)	1200	83.3	67.4

NLR, neutrophil-to-lymphocyte ratio; PLR, platelet-to-lymphocyte ratio; MLR, monocyte-to-lymphocyte ratio; SII, systemic immune inflammation; Se, sensitivity; Sp, specificity.

**Table 6:** The hazard ratio (HR) for MLR, NLR, PLR, and SII using the Cox proportional hazards regression model.

Characteristic	HR	*p*-Value
MLR ≥ median	1.27 (95% CI 0.45–3.6)	0.651
NLR ≥ median	1.57 (95% CI 0.61–4.08)	0.347
PLR ≥ median	1.31 (95% CI 0.48–3.53)	0.597
SII ≥ median	1.57 (95% CI 0.61–4.08)	0.347

NLR, neutrophil-to-lymphocyte ratio; PLR, platelet-to-lymphocyte ratio; MLR, monocyte-to-lymphocyte ratio; SII, systemic immune inflammation; HR, hazard ratio.

## Data Availability

The data used to support the findings of this study are available from the corresponding author upon request.

## Supplementary Materials

The following supporting information can be downloaded at: https://www.mdpi.com/article/10.3390/medicina59091523/s1, Table S1: STROBE Statement—checklist of items that should be included in reports of observational studies.
